# Miliary tuberculosis and central nervous system infection caused by hematogenous transmission from a primary subcutaneous tuberculous abscess: A case report

**DOI:** 10.1097/MD.0000000000044221

**Published:** 2025-09-05

**Authors:** Qiu-Shi Yang, Nian Wang, Shu-Song Ruan

**Affiliations:** a Department of Tuberculosis, Guiyang Public Health Clinical Center, Guiyang, Guizhou Province, China.

**Keywords:** abscess, case report, *cutaneous tuberculosis*, tuberculosis, tuberculous meningitis

## Abstract

**Rationale:**

We report an extremely rare case in which delayed diagnosis and treatment of *Mycobacterium tuberculosis* infection primarily involving the subcutaneous tissues of an extremity led to hematogenous dissemination of the infection and subsequent deterioration of the patient.

**Patient concerns:**

An 82-year-old man presented to our hospital with a painful mass on the right ankle for over a year, as well as persistent fever and shortness of breath for >14 days. He received piperacillin/tazobactam followed by meropenem, which failed to decrease his peak temperature.

**Diagnoses:**

After performing chest computed tomography, acid-fast staining of the abscess specimen, GeneXpert *M tuberculosis*/rifampin assay, and cerebrospinal fluid tests, the patient was diagnosed with miliary pulmonary tuberculosis and tuberculous meningitis hematogenously transmitted from a primary subcutaneous tuberculous abscess.

**Interventions:**

Isoniazid, rifampin, levofloxacin, linezolid, and ethambutol were administered through a nasogastric tube to treat the tuberculosis, and 5 mg of dexamethasone was administered to reduce the inflammatory response.

**Outcomes:**

The treatment was halted because of poor compliance, and the patient died of respiratory failure within 1 month of returning home.

**Lessons:**

We suggest tuberculosis screening or biopsy recommendations for chronic soft tissue swellings in high tuberculosis-burden areas, to avoid missed or delayed diagnosis.

## 1. Introduction

Tuberculosis (TB) remains a significant global health problem, and the World Health Organization estimates that 1 in 4 people worldwide are infected with *Mycobacterium tuberculosis*.^[1]^ According to the *Global Tuberculosis Report 2022*, 10.6 million people fell ill with TB in 2021. A total of 1.6 million people died from TB in 2021, making it the second leading cause of death worldwide that year.^[2,3]^ TB is a chronic granulomatous inflammatory disease caused by *M tuberculosis*, and it affects all organs of the body.^[4]^ Pulmonary TB is the most common presentation, while extrapulmonary TB accounts for 15% to 20% of known cases.^[5]^ Subcutaneous tuberculous abscesses are a rare manifestation of TB, accounting for 1% of extrapulmonary TB cases. They are primarily seen in cases of severe disseminated or osteoarticular TB^[4]^; hematogenous spread from primary subcutaneous TB from the extremities is very rare.^[6]^ Here, we report a case of miliary TB and central nervous system (CNS) infection due to the hematogenous dissemination of infection from a TB abscess on an ankle.

## 2. Case presentation

**Figure 1.**
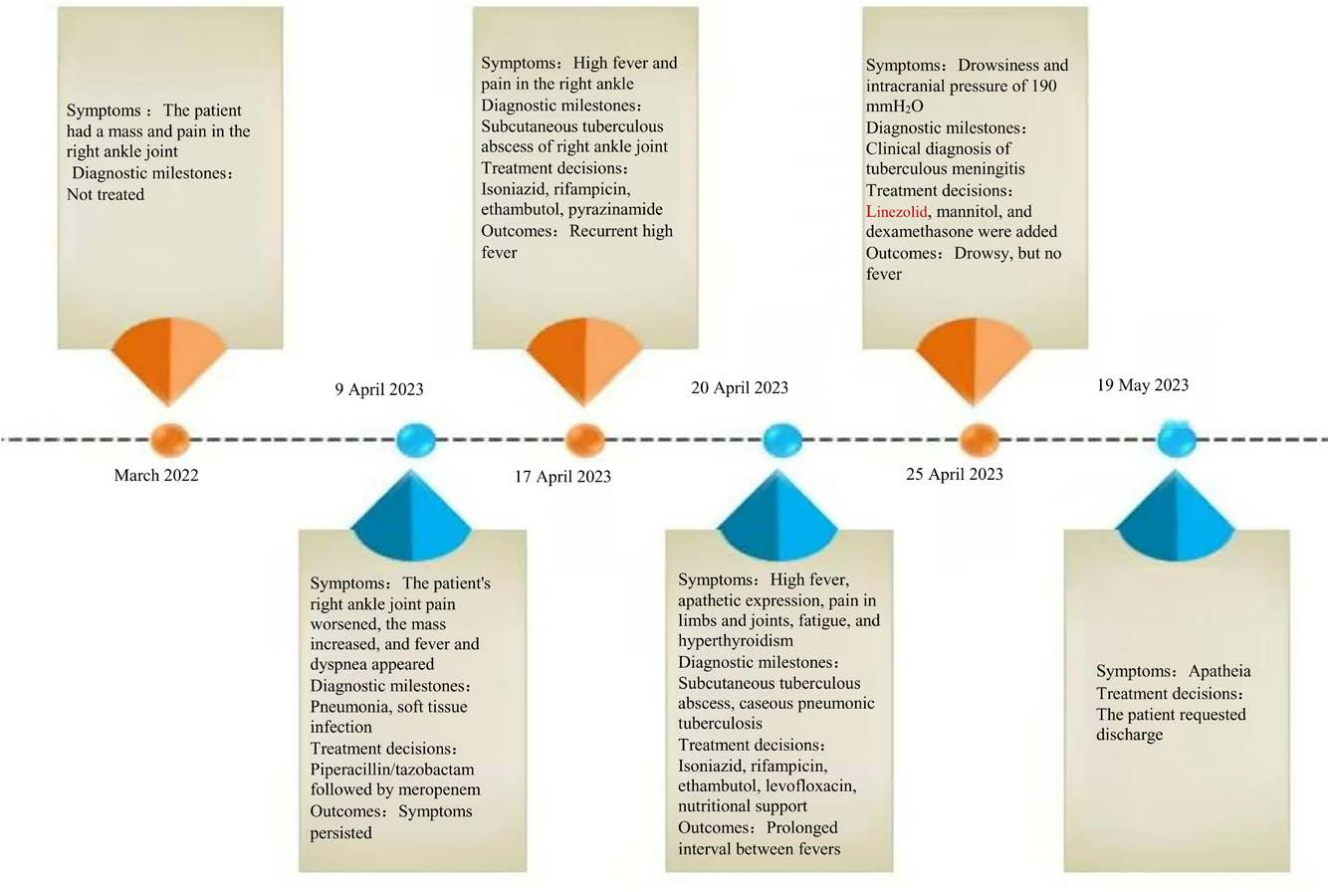
Chronological order of events.

An 82-year-old man was admitted to our hospital with complaints of a painful mass on his right ankle for more than a year, as well as fever and shortness of breath for more than 14 days. More than a year before his admission, the patient noticed right ankle pain of unknown cause, followed by the development of a mass that gradually increased in size. No fever, cough, sputum, hemoptysis, fatigue, or night sweats were reported. The patient was treated with multiple anti-infective therapies, all of which had poor efficacy. The patient developed a fever of unknown cause 14 days prior to admission, with a peak temperature of 39℃. The fever was intermittent but accompanied by dizziness, headache, chills, and shortness of breath after activity (Modified Medical Research Council Dyspnea Scale grade of 3), along with intense pain in the right ankle. The patient underwent anti-infective therapy with piperacillin/tazobactam followed by meropenem at an outpatient clinic; but the fever persisted without a decrease in peak body temperature. Acid-fast staining of the abscess specimen was positive, and the patient was diagnosed with a subcutaneous tuberculous abscess and transferred to the hospital’s TB department for further treatment. The chronological order of events is summarized in Figure 1. From the onset of illness, the patient demonstrated a lack of energy and appetite as well as poor-quality sleep. He had a history of untreated diabetes mellitus but denied any history of TB.

Personal history: The patient previously worked as a farmer and had a long history of agricultural work.

**Figure 2.**
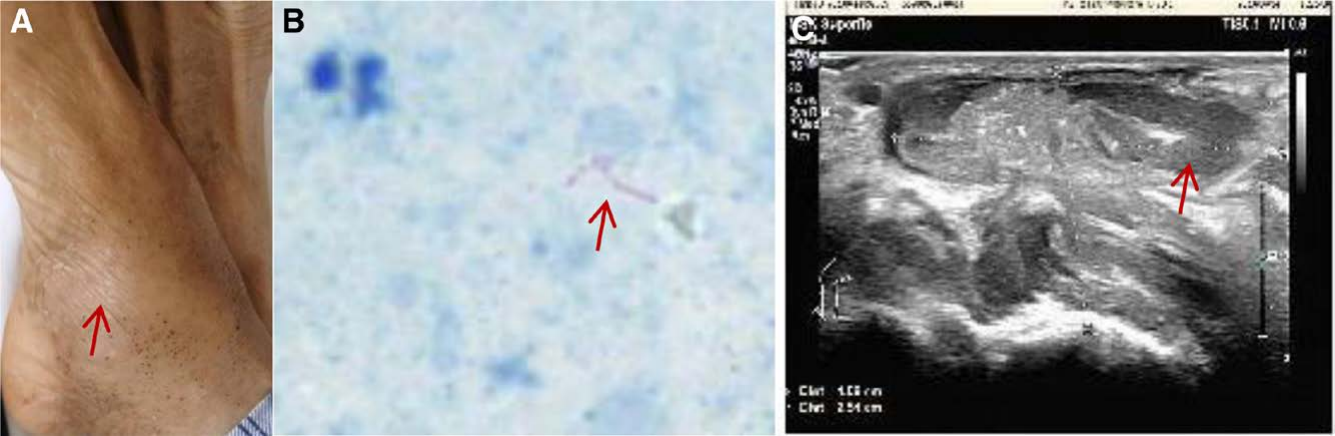
(A) Photo of the swollen right ankle (red arrow), (B) positive acid-fast staining (red arrow), and (C) color Doppler imaging of the right ankle, suggestive of a subcutaneous abscess.

Physical examination: The patient was emaciated and in low spirits. His body temperature was 37.6℃, and his systemic lymph nodes were not palpable. Coarse breathing sounds were heard, and moist rales were noted in the upper lungs bilaterally. No skin rupture was observed on the right ankle (Fig. 2A), although a palpable mass with obvious pressure pain was noted. No redness was noted in the area around the mass, and the skin temperature was normal. Physiological reflexes existed and pathological signs were not elicited.

**Figure 3.**
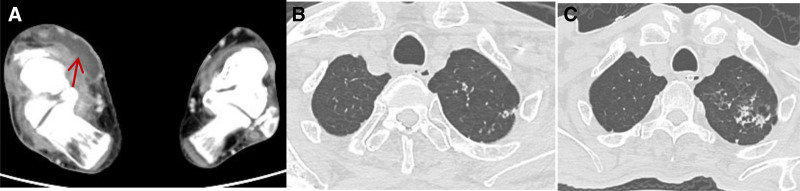
(A) Contrast-enhanced computed tomography (CT) scan of the right ankle indicating swelling of the soft tissue around the right talus (red arrow), (B) thoracic CT showing multiple hyperdense nodular shadows of varying sizes in both lungs, (C) pulmonary lesion progression following anti-tuberculosis treatment. CT = computed tomography.

Ancillary tests: The test results for routine blood, liver function, renal function, electrolytes, C-reactive protein, erythrocyte sedimentation rate, sepsis, galactomannan test, and serum (1,3)-β-d glucan were all normal. Blood cultures were negative for bacteria and human immunodeficiency virus, and glycosylated hemoglobin was 10.8%. Bacterial culture of the abscess specimen was negative, but acid-fast staining was positive (Fig. 2B). Immunoglobulin M class antibodies against respiratory pathogens were negative, as were the sputum antacid stain, solid culture for *M tuberculosis*, acid-fast staining for cerebrospinal fluid (CSF), GeneXpert for CSF, and CSF solid culture for *M tuberculosis*. The abscess specimen was positive for GeneXpert; the fecal occult blood test was also positive. Color Doppler imaging showed a flaky hypoechoic area in the talofibular, tibiofibular, and talocalcaneal spaces in the right medial malleolus (approximately 41 × 25 mm; Fig. 2C). Contrast-enhanced computed tomography (CT) of the right ankle revealed that the soft tissue around the right talus was markedly swollen with multiple striated bands and hypodense cystic shadows (Fig. 3A). Thoracic and total abdominal CT showed multiple hyperdense nodular shadows of varying sizes in both lungs, suggestive of subacute miliary TB. The bone structure was normal (Fig. 3B). Head CT suggested a small ischemic focus in the cerebral hemisphere around the central level of the lateral ventricle.

Diagnostic and therapeutic history: Although the patient initially received antibiotic therapy with piperacillin/tazobactam followed by meropenem, his recurrent high fever persisted with no improvement in the subcutaneous abscess. Acid-fast staining of the abscess specimen was positive, as was the nucleic acid testing for *M tuberculosis*, although the specimen did not show drug resistance to rifampin. Arterial blood gas analysis (supplemental oxygen, 3 L/min) revealed a pH of 7.47, partial pressure of oxygen of 69 mm Hg, partial pressure of CO_2_ of 50 mm Hg, bicarbonate level of 36.4 mmol/L, and a hemoglobin level of 28.5 g/L. Bacterial culture of the abscess specimen and cryptococcal antigen test of the blood and CSF were negative. *M tuberculosis*-related tests of the sputum, alveolar lavage fluid, and CSF were all negative. The CSF test results are presented in Table 1. The final diagnosis was as follows: subcutaneous tuberculous abscess, subacute miliary TB, tuberculous meningitis, diabetes, and type 2 respiratory failure. The abscess diagnosis was clear. US guidelines recommend levofloxacin as an alternative to pyrazinamide for intensified therapy in patients over 75 years of age.^[7]^ Owing to the patient’s advanced age, extreme weakness, and unbearable joint pain, pyrazinamide was not an option. Isoniazid, rifampin, levofloxacin, and ethambutol were administered through a nasogastric tube to treat the TB, and 5 mg of dexamethasone was administered to reduce the inflammatory response. No obvious abnormalities were observed in routine blood monitoring and liver and kidney function, as detailed in Table 2. Uncontrolled diabetes mellitus and malnutrition can affect the efficacy of anti-TB therapy, so insulin was used to lower blood glucose, and albumin and amino acids were used to improve the nutritional status. After treatment, the patient had good blood glucose control, his nutritional status improved, and the fever disappeared, but he had mental abnormalities during hospitalization. Physical examination suggested neck resistance. Skin TB was definitively diagnosed. Head CT showed a small ischemic focus in the cerebral hemisphere around the central level of the lateral ventricle. CSF testing showed increased albumin, decreased glucose and chloride, and a clinical diagnosis of tuberculous meningitis.^[8]^ According to Chinese guidelines for the diagnosis and treatment of TB of the CNS, Linezolid may benefit patients with severe disease; therefore, linezolid was added to the patient’s medication regimen to strengthen anti-TB therapy.^[9]^ Owing to the positive fecal occult blood test, the dexamethasone dose was not adjusted, to avoid inducing gastrointestinal bleeding. After proactive treatment, the tuberculous meningitis was significantly ameliorated, and the abscess in the right ankle resolved, but the lesions in the patient’s lungs progressed (Fig. 3C). The treatment was halted because of poor compliance, and the patient died of respiratory failure within 1 month of returning home.

**Table 1:** Biochemical markers following CSF anti-tuberculosis therapy.

	Pre-treatment			Post-treatment		
Date	21/4/2023	24/4/2023	27/4/2023	5/5/2023	11/5/2023	16/5/2023
CSF-Pro	186.7 mg/dL	174.7 mg/dL	122.3 mg/dL	119 mg/dL	96.1 mg/dL	79.4 mg/dL
Cl	109 mmol/L	115 mmol/L	111 mmol/L	111 mmol/L	113 mmol/L	109 mmol/L
GLU	6.31 mmol/L	5.17 mmol/L	3.95 mmol/L	1.65 mmol/L	3.6 mmol/L	2.5 mmol/L
ADA	16 U/L					

ADA = adenosine deaminase, Cl = chloride, CSF-Pro = cerebrospinal fluid protein, GLU = glucose.

**Table 2:** Laboratory results following anti-TB treatment.

Date	20/4/2023	26/4/2023	2/5/2023	9/5/2023	15/5/2023	18/5/2023
AST (U/L)	12	12	32	35	16	9
ALT (U/L)	12	8	17	51	15	12
TBIL (µmol/L)	16.2	8.4	9.4	10.9	11.6	10.8
ALB (g/L)	28.5	28.7	33.3	32.9	30.4	28.7
Cr (µmol/L)	38	43	38	34	33	37
UA (µmol/L)	180	118	56	47	49	93
HB (g/L)	134	110	107	117	107	109

ALB = albumin, ALT = alanine aminotransferase, AST = aspartate aminotransferase, Cr = creatinine, HB = hemoglobin, TBIL = total bilirubin, UA = uric acid.

## 3. Discussion

The clinical manifestations of cutaneous *M tuberculosis* infections are exceedingly diverse, including the formation of subcutaneous abscesses.^[6]^ The routes of infection include hematogenous dissemination, local or regional dissemination of deep infection, or direct implantation of *M tuberculosis* into the skin and/or soft tissue.^[6]^ Infections caused by the direct implantation of *M tuberculosis* into the skin are referred to as primary cutaneous TB, whereas subcutaneous tuberculous abscesses involve the reactivation of latent foci of *M tuberculosis* infection in various immunosuppressive conditions.^[10]^ Untreated primary cutaneous TB may resolve spontaneously in 12 months or more, or progress into disseminated disease via hematogenous transmission to other organs.^[6]^ Hematogenous dissemination from pulmonary lesions is the most common cause of cutaneous TB.^[1]^ However, hematogenous dissemination of primary cutaneous TB to other organs is very rare and is mostly seen in the extremities or faces of occupationally-exposed populations, such as butchers or farmers.^[6]^ We searched PubMed, Wanfang Data, China National Knowledge Infrastructure, and other databases using the terms “primary cutaneous,” “cutaneous tuberculosis,” and “subcutaneous tuberculosis,” but we found no reports of primary cutaneous TB. Disseminated TB involves 2 or more nonadjacent organs and is frequently observed in patients with immunodeficiency due to aging, malnutrition, malignancy, human immunodeficiency virus infection, or treatment with tumor necrosis factor-α inhibitors.^[11,12]^ Miliary TB is a distinct form of disseminated TB that can spread to the CNS, resulting in tuberculous meningitis.^[8,13]^ CNS TB is one of the least common but most devastating forms of human mycobacterial infection, and it has a high mortality rate.^[8]^ The low incidence, insidious onset, and lack of symptoms typically associated with subcutaneous tuberculous abscesses cause challenges and delays in diagnosis, which ultimately result in deterioration of the patient’s condition and increased TB transmission.^[14]^ Therefore, early diagnosis and treatment are crucial in these cases.^[11]^ CNS TB may present with fever and headache and progress to altered mentation and focal neurologic signs. The CSF formula typically shows lymphocytic pleocytosis and low glucose and high protein concentrations.^[9,15]^ Basal pool meningeal enhancement, hydrocephalus, cerebral infarction, and tuberculoma are the main imaging features of CNS TB, which can occur alone or in combination. Enlargement of the dura mater with or without tuberculoma is the most common sign of tuberculous meningitis, and it has high diagnostic specificity.^[9,15]^ Diagnosis relies on serial samples of CSF for smear and culture, combined with CSF PCR.^[9,15]^ Skin TB can be diagnosed by smear acid-fast staining, culture, PCR, or biopsy of specimens obtained by fine needle puncture. The presence of *M tuberculosis* confirms the diagnosis after suppurative abscess, fungal infection, and malignant tumor are excluded.^[15,16]^

Treatment options for subcutaneous TB abscesses include pharmacotherapy and surgery.^[12]^ Pharmacotherapy treatment involves intensive therapy with isoniazid, rifampin, pyrazinamide, and/or ethambutol for 2 months, followed by consolidation therapy with isoniazid and rifampin for an additional 4 months.^[17]^ Small abscesses may be treated with anti-TB drugs, but large abscesses are more likely to form fistulas or sinus tracts that require surgery, including drainage and surgical debridement. Regular anti-TB therapy should be administered before surgery, as failure to do so would increase the incidence of recurrent abscesses, unhealed lesions, or the subsequent formation of sinus tracts. Performing surgical incision, drainage, and resection of necrotic tissues for biopsy to obtain histopathologic specimens is both diagnostic and therapeutic.^[12,18]^ The principles of treatment for tuberculous meningitis are like those for pulmonary and cutaneous TB. Infections caused by non-drug-resistant TB bacteria can be treated with intensive pharmacotherapy, as described above, but with a longer consolidation therapy period (7–10 months).^[10]^ No standardized optimal treatment duration has been established, and treatment duration varies from country to country.^[19]^ Glucocorticoids are commonly used as an adjunctive therapy in the treatment of tuberculous meningitis to reduce inflammation and limit CNS damage, thereby improving prognosis.^[10]^

In the case presented here, the patient was a farmer who engaged in long-term agricultural work, which could have damaged the skin of his legs. *M tuberculosis* invades the skin, and the patient was in a high-risk group for *M tuberculosis* infection. Additional factors such as malnutrition, aging, and diabetes mellitus had weakened the patient’s immune system. The lesion initially developed in the subcutaneous tissue of his right ankle, which manifested as long-term pain at the lesion site. Broad-spectrum antibacterial therapy was not effective. The patient was confirmed to have *M tuberculosis* infection through acid-fast staining of the abscess specimen and the GeneXpert M tuberculosis/rifampin assay. In addition, the patient had recently developed fever, shortness of breath, and lethargy, for which laboratory results ruled out intracranial infection caused by fungi, viruses, or bacteria. His chest CT indicated disseminated TB, while CSF tests showed a significant increase in protein, significant decrease in sugar and chloride, and a clinical diagnosis of tuberculous meningitis.^[11]^ Therefore, we believe that the primary cutaneous tuberculous abscess resulted in TB and CNS infection. Both the subcutaneous abscess and tuberculous meningitis improved with anti-TB treatment; however, the lung lesions worsened, which is considered a paradoxical reaction to the anti-TB therapy. Paradoxical reactions are characterized by transient deterioration of existing infected lesions, onset of new lesions, or deterioration of existing TB lesions during anti-TB treatment, as determined by imaging.^[7,20]^ Such findings are attributed to paradoxical reactions only after excluding other possible causes, especially TB treatment failure from drug-resistant TB or another opportunistic disease.^[7]^ This may have been the result of an enhanced local inflammatory response following the mass destruction of *M tuberculosis* or an excessive immune response following control of the disseminated infection.^[20]^ Corticosteroids are the main drugs used in paradoxical response therapy and are safe and effective.^[20,21]^

## 4. Conclusion

We report an extremely rare case in which delayed diagnosis and treatment of *M tuberculosis* infection primarily involving the subcutaneous tissues of an extremity led to hematogenous dissemination of the infection and subsequent deterioration of the patient. Delays in diagnosis may contribute to TB spread, increase socioeconomic burden, and pose challenges for TB control, particularly for rural or resource-limited settings where such delays are more likely. We suggest TB screening or biopsy for chronic soft tissue swellings in high TB burden areas to prevent misdiagnosis and treatment delays.

## Acknowledgments

We would like to thank Editage (www.editage.cn) for English language editing.

## Author contributions

**Resources:** Nian Wang.

**Validation:** Shu-Song Ruan.

**Writing – original draft:** Qiu-Shi Yang.

**Writing – review & editing:** Shu-Song Ruan.
